# On the states of stress and strain adjacent to a crack in a strain-limiting viscoelastic body

**DOI:** 10.1177/1081286517709517

**Published:** 2017-05-30

**Authors:** Hiromichi Itou, Victor A Kovtunenko, Kumbakonam R Rajagopal

**Affiliations:** Department of Mathematics, Tokyo University of Science, Tokyo, Japan; Institute for Mathematics and Scientific Computing, University of Graz, NAWI Graz, Graz, Austria; Lavrentyev Institute of Hydrodynamics, Siberian Division of the Russian Academy of Sciences, Novosibirsk, Russia; Department of Mechanical Engineering, Texas A & M University, College Station, TX, USA

**Keywords:** Kelvin–Voigt viscoelastic solid, limiting small strain, crack, variational problem, generalized solution

## Abstract

The viscoelastic Kelvin–Voigt model is considered within the context of quasi-static deformations and generalized with respect to a nonlinear constitutive response within the framework of limiting small strain. We consider a solid possessing a crack subject to stress-free faces. The corresponding class of problems for strain-limiting nonlinear viscoelastic bodies with cracks is considered within a generalized formulation stated as variational equations and inequalities. Its generalized solution, relying on the space of bounded measures, is proved rigorously with the help of an elliptic regularization and a fixed-point argument.

## 1. Introduction

A class of nonlinear elasticity models allowing a nonlinear strain–stress response due to limiting small strain was introduced by Rajagopal [1] and developed in later works by the author. Strain-limiting constitutive relations expressing the linearized strain as a nonlinear function of the stress were studied [2]. This approach was developed further within the framework of viscoelasticity [3–5]. With regard to the use of such models to study the fracture of brittle materials [6, 7], a constitutive relation with the limiting small strain overcomes the inconsistency of infinite strains at the crack tip; this is the prediction of the linearized theory.

Within the context of nonlinear elasticity, Tarantino [8] studied the problem of the state of stress and strain at a crack tip using a neo-Hookean elastic model that obeys the Bell constraint and showed that the strain was bounded. However, the Bell constraint requires the sum of the principal stresses to add up to three; this would not allow a body to undergo a simple shear motion! Moreover, Bell found the constraint to hold in a body undergoing inelastic response and did not establish the constraint in the elastic regime. Horgan and Saccomandi [9] studied the state of strain at a crack tip using a modification of the limited chain extensibility model introduced by Gent [10]. Not surprisingly, Horgan and Saccomandi [9] found that there are no singularities at the crack tip, as the strain is limited a priori in their model. Nonetheless, the result is significant in that it is one of the earliest studies in elasticity to demonstrate that a singularity does not necessarily have to be present at a crack tip.

In this work, we provide a rigorous mathematical background for viscoelastic problems with limiting small strain in domains with cracks. This is a main feature of our model; that is, boundedness (smallness) of strain is required, but there are no constraints about stress. Namely, our model covers stress concentration phenomena even under infinitesimal strain.

The classic Kelvin–Voigt viscoelastic model whose mechanical analog is a linearized elastic spring in parallel with a viscous dashpot (see, e.g., Truesdell and Noll [11], Section 41) can be written with respect to the linearized strain ε, the time rate of the linearized strain $\dot{\varepsilon }$ (where the dot stands for the time derivative), and the stress σ in the linearized version, as follows:

(1) $$\varepsilon +\alpha \;\dot{\varepsilon }=\beta \sigma ,$$

with material constants α>0 and β>0 (where 1∕β is the shear modulus and α∕(2β) is the viscosity). It is worth observing that, as in the case of the linearized elastic model, the linearized Kelvin–Voigt model given here is not frame-indifferent. We ought to view it as the linearized approximation that is valid when the displacement gradients are small. In the framework of limiting small strain, we generalize the linear Kelvin–Voigt model (equation (1)) to the following nonlinear relations:

(2) $$\varepsilon +\alpha \;\dot{\varepsilon }=F\left(\sigma \right),\;∥F\left(\sigma \right)∥\leq M_{1},$$

where constant $M_{1}>0$, and F stands for a response function.

Since $∥F\left(\sigma \right)∥\leq M_{1}$, we have two-sided bounds $-M_{1}\leq F_{\mathrm{ij}}\left(\sigma \right)\leq M_{1}$ component-wise. On applying the upper bound $F_{\mathrm{ij}}\left(\sigma \right)\leq M_{1}$ to the equality in the Kelvin–Voigt model (equation (1)), rewriting it in the following form:

$$\frac{d}{\mathrm{dt}}\left(\varepsilon_{\mathrm{ij}}-M_{1}\right)\leq -\frac{1}{\alpha }\left(\varepsilon_{\mathrm{ij}}-M_{1}\right),$$

and integrating the latter inequality, we derive the upper estimate:

$$\varepsilon_{\mathrm{ij}}\left(t\right)-M_{1}\leq \left(\varepsilon_{\mathrm{ij}}\left(0\right)-M_{1}\right)e^{-t∕\alpha }.$$

Henceforth, if $\varepsilon_{\mathrm{ij}}\left(0\right)\leq M_{1}$ at the initial time *t*=0, then $\varepsilon_{\mathrm{ij}}\left(t\right)\leq M_{1}$ for all times t>0. Analogously, using the lower bound $F_{\mathrm{ij}}\left(\sigma \right)\geq -M_{1}$, such that

$$\frac{d}{\mathrm{dt}}\left(\varepsilon_{\mathrm{ij}}+M_{1}\right)\geq -\frac{1}{\alpha }\left(\varepsilon_{\mathrm{ij}}+M_{1}\right),$$

after integration we obtain the lower estimate

$$\varepsilon_{\mathrm{ij}}\left(t\right)+M_{1}\geq \left(\varepsilon_{\mathrm{ij}}\left(0\right)+M_{1}\right)e^{-t∕\alpha },$$

hence $\varepsilon_{\mathrm{ij}}\left(t\right)\geq -M_{1}$ when $\varepsilon_{\mathrm{ij}}\left(0\right)\geq -M_{1}$. This follows the uniform boundedness $|\varepsilon_{\mathrm{ij}}\left(t\right)|\leq M_{1}$ for t>0 provided by $|\varepsilon_{\mathrm{ij}}\left(0\right)|\leq M_{1}$. As a consequence of the equality

$${\dot{\varepsilon }}_{\mathrm{ij}}=\frac{1}{\alpha }\left(F_{\mathrm{ij}}\left(\sigma \right)-\varepsilon_{\mathrm{ij}}\right),$$

we have that

$$|{\dot{\varepsilon }}_{\mathrm{ij}}\left(t\right)|\leq \frac{2}{\alpha }M_{1}$$

is also bounded uniformly for t≥0. Moreover, ε(t) remains small when the constant *M*_1_ and the initial deformation ε(0) are prescribed to be small.

An example of the function to keep in mind is (see Rajagopal [2]):

(3) $$F\left(\sigma \right)=\frac{\beta \sigma }{{\left(1+\kappa ∥\sigma {∥}^{s}\right)}^{1∕s}},\;\beta ,\kappa ,s>0,$$

which is uniformly bounded with $M_{1}=\beta ∕\left(\kappa^{1∕s}\right)$. We note that, if the parameter κ tends to zero in equation (3), then the limiting small strain (equation (2)) turns into the classical Kelvin–Voigt model (equation (1)).

Analysis of the nonlinear model (equation (2)) obeys the problematic issue of singular stress existing in the space of bounded measures. This mathematical difficulty was attacked in Beck et al. [12], Bulicek et al. [13], and other works by these authors, providing us with special situations when the measure is localized at the Neumann boundary. In the linear case of the Kelvin–Voigt model (equation (1)), contact viscoelastic problems subject to Signorini boundary conditions were proved rigorously in Shillor et al. [14], Chapter 8, based on the theory of set-valued maximal monotone operators.

Another specific feature of our consideration concerns the presence of a crack in the solid. In the mathematical literature, there are very few results concerning crack problems in viscoelasticity. Using the Laplace transform, the linear viscoelastic problem with crack was investigated [15, 16]. To study the related creep problems for cracks subject to contacting faces, a technique using elliptic regularization and penalization was developed [17, 18]. The variational theory of nonlinear crack problems with contact conditions was established [19–23].

If the parameter α is set to be zero in equation (2), the nonlinear viscoelastic constitutive expression reduces to the nonlinear elastic constitutive expression. In this case, the problem with limiting small strain and crack subject to contacting faces was discussed previously [24]. In this paper, we extend this concept to the nonlinear viscoelastic body with a stress-free crack. A generalized formulation of the problem requires variational equations and inequalities and relies on the space of bounded measures. Its solvability is proved based on an elliptic regularization and the Schauder–Tychonoff fixed-point theorem.

## 2. Formulation of the nonlinear viscoelastic problem with crack

In the Euclidean space ${\mathbb{R}}^{d}$ equipped with the inner product $x\cdot n=\sum_{i=1}^{d}x_{i}n_{i}$ and the associated vector norm $∥x∥=\sqrt{x\cdot x}$ for vectors $x=\left(x_{1},\ldots ,x_{d}\right)$ and $n=\left(n_{1},\ldots ,n_{d}\right)$, where the dimension is either *d*=2 or *d*=3, we consider a domain $\Omega \subset {\mathbb{R}}^{d}$. Let its boundary ∂Ω be a (d−1) -dimensional Lipschitz manifold obeying the outward normal vector *n*. Let the boundary consist of mutually disjoint Neumann and Dirichlet parts, $\Gamma_{N}$ and $\Gamma_{D}$, respectively, such that $\partial \Omega =\bar{\Gamma_{N}}\cup \bar{\Gamma_{D}}$ and $\Gamma_{D}\neq \emptyset$.

We consider the crack $\Gamma_{c}\subset \Omega$ as a part of a (d−1) -dimensional oriented manifold splitting Ω into two domains with Lipschitz boundaries. A normal vector *n* chosen at $\Gamma_{c}$ determines two opposite crack faces: the positive $\Gamma_{c}^{+}$ in the direction of *n*, and the negative $\Gamma_{c}^{-}$, corresponding to (−n). We associate $\Omega_{c}:=\Omega \backslash \Gamma_{c}$ with the spatial domain with crack. For time variables t∈(0,τ) and τ∈(0,T), with some T>0 fixed, the time-cylinder is denoted by $Q_{c}^{\tau }:=\left(0,\tau \right)\times \Omega_{c}$ and $Q_{c}^{T}:=\left(0,T\right)\times \Omega_{c}$, respectively.

We introduce the space $\mathrm{Sym}\left({\mathbb{R}}^{d\times d}\right)$ of symmetric *d* -by-*d* tensors $\sigma ={\left\{\sigma_{\mathrm{ij}}\right\}}_{i,j=1}^{d}$ and $\varepsilon ={\left\{\varepsilon_{\mathrm{ij}}\right\}}_{i,j=1}^{d}$, equipped with the inner product $\sigma :\varepsilon =\sum_{i,j=1}^{d}\sigma_{\mathrm{ij}}\varepsilon_{\mathrm{ij}}$ and the associated matrix norm $∥\sigma ∥=\sqrt{\sigma :\sigma }$. In this space, the viscoelastic response can be expressed by a map:

(4) $$F:\;\mathrm{Sym}\left({\mathbb{R}}^{d\times d}\right)\mapsto \mathrm{Sym}\left({\mathbb{R}}^{d\times d}\right),\;F\left(0\right)=0.$$

The following properties of the tensor-valued function $F={\left\{F_{\mathrm{ij}}\right\}}_{i,j=1}^{d}$ in equation (4) are assumed. Let constants $M_{1},M_{3}>0$ and $M_{2}\geq 0$ exist, such that for all $\sigma ,\bar{\sigma }\in \mathrm{Sym}\left({\mathbb{R}}^{d\times d}\right)$ the following hold:

(5a) $$∥F\left(\sigma \right)∥\leq M_{1},$$

(5b) $$\left(F\left(\sigma \right)-F\left(\bar{\sigma }\right)\right):\left(\sigma -\bar{\sigma }\right)\geq 0,$$

(5c) $$s\mapsto F\left(\sigma +s\bar{\sigma }\right):\bar{\sigma }\;\mathrm{is continuous at}\;s=0,$$

(5d) $$-M_{2}+M_{3}\overset{d}{\underset{i,j=1}{\sum }}|\sigma_{\mathrm{ij}}|\leq F\left(\sigma \right):\sigma .$$

Here the properties imply the uniform boundedness (equation (5a)), monotony (equation (5b)), hemi-continuity (equation (5c)), and semi-coercivity (equation (5d)). In particular, for the conceptual example of equation (3), the property of equation (5c) is a consequence of the Lipschitz continuity $\left(F\left(\sigma \right)-F\left(\bar{\sigma }\right)\right):\left(\sigma -\bar{\sigma }\right)\leq 2\beta ∥\sigma -\bar{\sigma }{∥}^{2}$, and equation (5d) holds with the constants $M_{2}=\beta ∕\left(\kappa^{2∕s}c_{s}\right)$ and $M_{3}=\beta ∕\left(d\kappa^{1∕s}c_{s}\right)$, where $c_{s}=2^{1∕s-1}$ for s∈(0,1) and $c_{s}=1$ for s≥1 [13]. The hypotheses of equation (5) are needed from technical requirements; the physical meaning for the example of the response function F of equation (3) is demonstrated in Rajagopal [2].

Let vector-valued functions for the body force $f\left(t,x\right)=\left(f_{1},\ldots ,f_{d}\right)\in C\left(\left[0,T\right];L^{2}\left(\Omega_{c};{\mathbb{R}}^{d}\right)\right)$ and the boundary traction $g\left(t,x\right)=\left(g_{1},\ldots ,g_{d}\right)\in C\left(\left[0,T\right];L^{2}\left(\Gamma_{N};{\mathbb{R}}^{d}\right)\right)$ be given. We introduce an auxiliary quasi-static linear elasticity problem under Hooke’s law, for instance $\varepsilon \left(u^{E}\right)=\sigma^{E}$, where $\sigma^{E},\varepsilon \left(u^{E}\right)\in \mathrm{Sym}\left({\mathbb{R}}^{d\times d}\right)$, and the linearized strain is defined by the formula

(6) $$\varepsilon_{\mathrm{ij}}\left(u\right):=\frac{1}{2}\left(\frac{\partial }{\partial x_{j}}u_{i}+\frac{\partial }{\partial x_{i}}u_{j}\right),\;i,j=1,\ldots ,d.$$

The elastic solution implies the displacement $u^{E}\left(t,x\right)\in C\left(\left[0,T\right];H^{1}\left(\Omega_{c};{\mathbb{R}}^{d}\right)\right)$, such that $u^{E}=0$ at $\left(0,T\right)\times \Gamma_{D}$ and the Cauchy stress $\sigma^{E}\left(t,x\right)\in C(\left[0,T\right];L^{2}\left(\Omega_{c};\mathrm{Sym}\left({\mathbb{R}}^{d\times d}\right)\right)$, which satisfy

(7) $$\int_{\Omega_{c}}\sigma^{E}:\varepsilon \left(\bar{u}\right)\;\mathrm{dx}=\int_{\Omega_{c}}f\cdot \bar{u}\;\mathrm{dx}+\int_{\Gamma_{N}}g\cdot \bar{u}\;dS_{x},\;t\in \left(0,T\right),$$

for all test functions $\bar{u}\in H^{1}\left(\Omega_{c};{\mathbb{R}}^{d}\right)$ such that $\bar{u}=0$ at $\Gamma_{D}$. Without loss of generality, we assume that the initial state $u^{0}\left(x\right)=\left(u_{1}^{0},\ldots ,u_{d}^{0}\right)\in H^{1}\left(\Omega_{c};{\mathbb{R}}^{d}\right)$ is given such that the compatibility condition holds:

(8) $$u^{0}=u^{E}\left(0,\;\cdot \;\right)\;\mathrm{in}\;\Omega_{c}.$$

We look for vectors for the displacement in the viscoelasticity problem, namely $u\left(t,x\right)=\left(u_{1},\ldots ,u_{d}\right)$ with the velocity $\dot{u}\left(t,x\right)=\left({\dot{u}}_{1},\ldots ,{\dot{u}}_{d}\right)$, tensors of the Cauchy–Green strain $\varepsilon \left(t,x\right)\in \mathrm{Sym}\left({\mathbb{R}}^{d\times d}\right)$ and the Cauchy stress $\sigma \left(t,x\right)\in \mathrm{Sym}\left({\mathbb{R}}^{d\times d}\right)$, which satisfy the following quasi-static initial boundary value problem, written component-wise for i,j=1,…,d as follows:

(9a) $$\begin{array}{cc} -\overset{d}{\underset{j=1}{\sum }}\frac{\partial }{\partial x_{j}}\sigma_{\mathrm{ij}} & =f_{i}\;\mathrm{in}\;Q_{c}^{T}, \end{array}$$

(9b) $$\begin{array}{cc} \varepsilon_{\mathrm{ij}}\left(u\right)+\alpha \varepsilon_{\mathrm{ij}}\left(\dot{u}\right) & =F_{\mathrm{ij}}\left(\sigma \right)\;\mathrm{in}\;Q_{c}^{T}, \end{array}$$

(9c) $$\begin{array}{cc} u_{i}\left(0,\;\cdot \;\right) & =u_{i}^{0}\;\mathrm{in}\;\Omega_{c}, \end{array}$$

(9d) $$\begin{array}{cc} u_{i} & =0\;\mathrm{on}\;\left(0,T\right)\times \Gamma_{D}, \end{array}$$

(9e) $$\begin{array}{cc} {\left(\sigma_{n}\right)}_{i} & =g_{i}\;\mathrm{on}\;\left(0,T\right)\times \Gamma_{N}, \end{array}$$

(9f) $$\begin{array}{cc} {\left(\sigma_{n}\right)}_{i} & =0\;\mathrm{on}\;\left(0,T\right)\times \Gamma_{c}^{\pm }, \end{array}$$

where $\sigma_{n}:={\left\{\sum_{j=1}^{d}\sigma_{\mathrm{ij}}n_{j}\right\}}_{i=1}^{d}$ represents the boundary stress vector, and the linearized strain is defined in equation (6). The system of equations (9) constitutes the equilibrium equation (9a) and the constitutive equations (9b), which are endowed with the initial (equation (9c)) and the mixed Dirichlet (equation (9d)), Neumann-type (equation (9e)), and stress-free crack (equation (9f)) boundary conditions.

Using the standard approach, forming the scalar product of the equations (9a) with a vector $\bar{u}\left(x\right)=\left({\bar{u}}_{1},\ldots ,{\bar{u}}_{d}\right)$, after integration by parts over $\Omega_{c}$ with the help of the boundary conditions of equations (9e) and (9f), and forming the scalar product of equation (9b) with a tensor $\bar{\sigma }\left(x\right)\in \mathrm{Sym}\left({\mathbb{R}}^{d\times d}\right)$, we arrive at a variational formulation of the problem of equation (9): Find the displacement $u\in C\left(\left[0,T\right];H^{1}\left(\Omega_{c};{\mathbb{R}}^{d}\right)\right)$ such that equations (9c) and (9d) hold, the strain $\varepsilon \left(u\right)\in C^{1}(\left[0,T\right];L^{\infty }\left(\Omega_{c};\mathrm{Sym}\left({\mathbb{R}}^{d\times d}\right)\right)$, and the stress $\sigma \in C(\left[0,T\right];L^{2}\left(\Omega_{c};\mathrm{Sym}\left({\mathbb{R}}^{d\times d}\right)\right)$, which satisfy two variational equations:

(10a) $$\begin{array}{cc} \int_{\Omega_{c}}\sigma :\varepsilon \left(\bar{u}\right)\;\mathrm{dx} & =\int_{\Omega_{c}}f\cdot \bar{u}\;\mathrm{dx}+\int_{\Gamma_{N}}g\cdot \bar{u}\;dS_{x},\;t\in \left(0,T\right), \end{array}$$

(10b) $$\begin{array}{cc} \int_{\Omega_{c}}\left(\varepsilon \left(u\right)+\alpha \varepsilon \left(\dot{u}\right)-F\left(\sigma \right)\right):\bar{\sigma }\;\mathrm{dx} & =0,\;t\in \left(0,T\right), \end{array}$$

for all test functions $\bar{u}\in H^{1}\left(\Omega_{c};{\mathbb{R}}^{d}\right)$, such that $\bar{u}=0$ at $\Gamma_{D}$ and $\bar{\sigma }\in L^{2}\left(\Omega_{c};\mathrm{Sym}\left({\mathbb{R}}^{d\times d}\right)\right)$. The inclusion of ε(u) and $\varepsilon \left(\dot{u}\right)$ in the $L^{\infty }$ -space is based on the constitutive relation of equation (9b) and the uniform bound of equation (5a) of F by the argument given in the introduction. If the stress $\sigma \in L^{2}\left(\Omega_{c};\mathrm{Sym}\left({\mathbb{R}}^{d\times d}\right)\right)$, then the normal stress $\sigma_{n}$ in equations (9e) and (9f) can be discussed in terms of Green’s formula in the dual space $H^{-1∕2}\left(\Gamma_{N};{\mathbb{R}}^{d}\right)\times H^{-1∕2}\left(\Gamma_{c};{\mathbb{R}}^{d}\right)$ at the boundary (see Khludnev and Kovtunenko [18], Section 1.1.7, for details).

It is important to emphasize that the nonlinearity of F does not allow us to apply standard existence theorems to the weak formulation of equation (10). Instead, in the following, we introduce a generalized formulation of the problem.

From equation (10b) rewritten with the test function $\bar{\sigma }-\sigma$, we have

(11) $$\int_{\Omega_{c}}\left(\varepsilon \left(u\right)+\alpha \varepsilon \left(\dot{u}\right)\right):\left(\bar{\sigma }-\sigma \right)\;\mathrm{dx}=\int_{\Omega_{c}}F\left(\sigma \right):\left(\bar{\sigma }-\sigma \right)\;\mathrm{dx}.$$

Since the Dirichlet condition of equation (9d) holds, it can be differentiated with respect to time, then $\dot{u}=0$ at $\left(0,T\right)\times \Gamma_{D}$, and $\bar{u}=u+\alpha \dot{u}$ can be substituted as a test function into equation (10a). It follows that

(12) $$\int_{\Omega_{c}}\sigma :\left(\varepsilon \left(u\right)+\alpha \varepsilon \left(\dot{u}\right)\right)\;\mathrm{dx}=\int_{\Omega_{c}}f\cdot \left(u+\alpha \dot{u}\right)\;\mathrm{dx}+\int_{\Gamma_{N}}g\cdot \left(u+\alpha \dot{u}\right)\;dS_{x}.$$

Using the monotony of F in equation (5b), summation of equations (11) and (12) results in the inequality:

(13) $$\begin{array}{c} \int_{\Omega_{c}}\left(\varepsilon \left(u\right)+\alpha \varepsilon \left(\dot{u}\right)\right):\bar{\sigma }\;\mathrm{dx}=\int_{\Omega_{c}}F\left(\sigma \right):\left(\bar{\sigma }-\sigma \right)\;\mathrm{dx}+\int_{\Omega_{c}}f\cdot \left(u+\alpha \dot{u}\right)\;\mathrm{dx}+\int_{\Gamma_{N}}g\cdot \left(u+\alpha \dot{u}\right)\;dS_{x}\\ \leq \int_{\Omega_{c}}F\left(\bar{\sigma }\right):\left(\bar{\sigma }-\sigma \right)\;\mathrm{dx}+\int_{\Omega_{c}}f\cdot \left(u+\alpha \dot{u}\right)\;\mathrm{dx}+\int_{\Gamma_{N}}g\cdot \left(u+\alpha \dot{u}\right)\;dS_{x}, \end{array}$$

which is advantageous, since the terms F(σ):σ in equation (11) and $\sigma :\varepsilon \left(u+\alpha \dot{u}\right)$ in equation (12) create problems with regard to establishing estimates within the weak formulation. In fact, only an *L*^1^ -estimate of the stress tensor is available by the lower bound in equation (5d).

Therefore, using the compact embedding of $L^{1}\left(\Omega_{c}\right)$ in the space of bounded measures $M^{1}\left(\Omega_{c}\right)$, which is dual to the space $C_{c}\left(\Omega_{c}\right)$ of continuous functions with the compact support in $\Omega_{c}$, we define a generalized solution in larger function spaces: Find the displacement $u\in C\left(\left[0,T\right];H^{1}\left(\Omega_{c};{\mathbb{R}}^{d}\right)\right)$ that satisfies the initial (equation (9c)) and Dirichlet (equation (9d)) conditions, the strain $\varepsilon \left(u\right)\in C^{1}(\left[0,T\right];L^{2}\left(\Omega_{c};\mathrm{Sym}\left({\mathbb{R}}^{d\times d}\right)\right)$, and the stress $\sigma \in C(\left[0,T\right];M^{1}\left(\Omega_{c};\mathrm{Sym}\left({\mathbb{R}}^{d\times d}\right)\right)$, which fulfill the variational equation (10a) and the inequality (13) in the following sense:

(14a) $$\int_{\Omega_{c}}\sigma :\varepsilon \left(\bar{u}\right)\;\mathrm{dx}=\int_{\Omega_{c}}f\cdot \bar{u}\;\mathrm{dx}+\int_{\Gamma_{N}}g\cdot \bar{u}\;dS_{x},\;t\in \left(0,T\right),$$

(14b) $$\begin{array}{c} \int_{\Omega_{c}}\left(\varepsilon \left(u\right)+\alpha \varepsilon \left(\dot{u}\right)\right):\bar{\sigma }\;\mathrm{dx}\leq \int_{\Omega_{c}}F\left(\bar{\sigma }\right):\left(\bar{\sigma }-\sigma \right)\;\mathrm{dx}+\int_{\Omega_{c}}f\cdot \left(u+\alpha \dot{u}\right)\;\mathrm{dx}+\int_{\Gamma_{N}}g\cdot \left(u+\alpha \dot{u}\right)\;dS_{x},\\ t\in \left(0,T\right), \end{array}$$

for all test functions $\bar{u}\in H^{1}\left(\Omega_{c};{\mathbb{R}}^{d}\right)$, such that $\bar{u}=0$ at $\Gamma_{D}$ and $\varepsilon \left(\bar{u}\right),\bar{\sigma }\in C_{c}\left(\Omega_{c};\mathrm{Sym}\left({\mathbb{R}}^{d\times d}\right)\right)$. By this, the “integrals” including σ in equation (14) are understood as the duality between $M^{1}\left(\Omega_{c};\mathrm{Sym}\left({\mathbb{R}}^{d\times d}\right)\right)$ and $C_{c}\left(\Omega_{c};\mathrm{Sym}\left({\mathbb{R}}^{d\times d}\right)\right)$.

In the following, we prove the solvability of the generalized formulation (equation (14)).

## 3. Existence theorems

Our approach is based on an elliptic regularization of the limiting small strain problem.

For a small fixed parameter δ>0, we set the regularized problem: Find $u^{\delta }\in C\left(\left[0,T\right];H^{1}\left(\Omega_{c};{\mathbb{R}}^{d}\right)\right)$, $\varepsilon \left(u^{\delta }\right)\in C^{1}(\left[0,T\right];L^{2}\left(\Omega_{c};\mathrm{Sym}\left({\mathbb{R}}^{d\times d}\right)\right)$, and $\sigma^{\delta }\in C(\left[0,T\right];L^{2}\left(\Omega_{c};\mathrm{Sym}\left({\mathbb{R}}^{d\times d}\right)\right)$, such that

(15a) $$u^{\delta }\left(0,\;\cdot \;\right)=u^{0}\;\mathrm{in}\;\Omega_{c},$$

(15b) $$u^{\delta }=0\;\mathrm{on}\;\left(0,T\right)\times \Gamma_{D},$$

(15c) $$\int_{\Omega_{c}}\sigma^{\delta }:\varepsilon \left(\bar{u}\right)\;\mathrm{dx}=\int_{\Omega_{c}}f\cdot \bar{u}\;\mathrm{dx}+\int_{\Gamma_{N}}g\cdot \bar{u}\;dS_{x},\;t\in \left(0,T\right),$$

(15d) $$\int_{\Omega_{c}}\left(\varepsilon \left(u^{\delta }\right)+\alpha \varepsilon \left({\dot{u}}^{\delta }\right)-F\left(\sigma^{\delta }\right)-\delta \sigma^{\delta }\right):\bar{\sigma }\;\mathrm{dx}=0,\;t\in \left(0,T\right),$$

for all test functions $\bar{u}\in H^{1}\left(\Omega_{c};{\mathbb{R}}^{d}\right)$, such that $\bar{u}=0$ at $\Gamma_{D}$, and $\bar{\sigma }\in L^{2}\left(\Omega_{c};\mathrm{Sym}\left({\mathbb{R}}^{d\times d}\right)\right)$.

**Theorem 1**. *Let the tensor-function of the response* F *defined in equation (4) satisfy the properties of equation (5). For fixed* δ>0, *there exists a solution* $\left(u^{\delta },\varepsilon \left(u^{\delta }\right),\sigma^{\delta }\right)$ *to the regularized problem of equation (15). Depending on the data f and g, by means of an elastic stress* $\sigma^{E}$ *from equation (7), the solution satisfies the following a-priori estimates*:

(16a) $$\int_{\Omega_{c}}\left(\frac{\delta }{2}∥\sigma^{\delta }{∥}^{2}+M_{3}\overset{d}{\underset{i,j=1}{\sum }}|\sigma_{\mathrm{ij}}^{\delta }|\right)\;\mathrm{dx}\leq \int_{\Omega_{c}}\left(M_{2}+\frac{\delta }{2}∥\sigma^{E}{∥}^{2}+M_{1}∥\sigma^{E}∥\right)\;\mathrm{dx}=:K_{1},$$

(16b) $$\frac{1}{2}\int_{Q_{c}^{T}}∥\varepsilon \left(u^{\delta }\right){∥}^{2}\;\mathrm{dx}\mathrm{dt}+\frac{\alpha }{2}\underset{t\in \left[0,T\right]}{\max}\int_{\Omega_{c}}∥\varepsilon \left(u^{\delta }\right){∥}^{2}\;\mathrm{dx}\leq \frac{\alpha }{2}\int_{\Omega_{c}}∥\varepsilon \left(u^{0}\right){∥}^{2}\;\mathrm{dx}+M_{1}^{2}|Q_{c}^{T}|+2\mathrm{\delta T}K_{1}=:K_{2},$$

(16c) $$\frac{\alpha^{2}}{4}\int_{\Omega_{c}}∥\varepsilon \left({\dot{u}}^{\delta }\right){∥}^{2}\;\mathrm{dx}\leq M_{1}^{2}|\Omega_{c}|+2\delta K_{1}+\frac{2K_{2}}{\alpha }=:K_{3}.$$

**Proof.** We consider the following linearization of the problem of equation (15): Starting with a given initialization $\sigma^{\left(0\right)}\in C(\left[0,T\right];L^{2}\left(\Omega_{c};\mathrm{Sym}\left({\mathbb{R}}^{d\times d}\right)\right)$, for every m∈ℕ, find $u^{\left(m\right)}\in C\left(\left[0,T\right];H^{1}\left(\Omega_{c};{\mathbb{R}}^{d}\right)\right)$, $\varepsilon \left(u^{\left(m\right)}\right)\in C^{1}(\left[0,T\right];L^{2}\left(\Omega_{c};\mathrm{Sym}\left({\mathbb{R}}^{d\times d}\right)\right)$, and $\sigma^{\left(m\right)}\in C(\left[0,T\right];L^{2}\left(\Omega_{c};\mathrm{Sym}\left({\mathbb{R}}^{d\times d}\right)\right)$, such that

(17a) $$u^{\left(m\right)}\left(0,\;\cdot \;\right)=u^{0}\;\mathrm{in}\;\Omega_{c},$$

(17b) $$u^{\left(m\right)}=0\;\mathrm{on}\;\left(0,T\right)\times \Gamma_{D},$$

(17c) $$\int_{\Omega_{c}}\sigma^{\left(m\right)}:\varepsilon \left(\bar{u}\right)\;\mathrm{dx}=\int_{\Omega_{c}}f\cdot \bar{u}\;\mathrm{dx}+\int_{\Gamma_{N}}g\cdot \bar{u}\;dS_{x},\;t\in \left(0,T\right),$$

(17d) $$\int_{\Omega_{c}}\left(\varepsilon \left(u^{\left(m\right)}\right)+\alpha \varepsilon \left({\dot{u}}^{\left(m\right)}\right)-\delta \sigma^{\left(m\right)}\right):\bar{\sigma }\;\mathrm{dx}=\int_{\Omega_{c}}F\left(\sigma^{\left(m-1\right)}\right):\bar{\sigma }\;\mathrm{dx},\;t\in \left(0,T\right),$$

for all test functions $\bar{u}\in H^{1}\left(\Omega_{c};{\mathbb{R}}^{d}\right)$, such that $\bar{u}=0$ at $\Gamma_{D}$, and $\bar{\sigma }\in L^{2}\left(\Omega_{c};\mathrm{Sym}\left({\mathbb{R}}^{d\times d}\right)\right)$. At every iterate *m*, the system of equation (17) implies a linear problem for the viscoelastic response, which obeys a solution $\left(u^{\left(m\right)},\varepsilon \left(u^{\left(m\right)}\right),\sigma^{\left(m\right)}\right)$ according to the general theory of linear operators, (see, e.g., Ladyzhenskaya [25], Chapter 8).

To guarantee a limit as m→∞ in equation (17), we will derive uniform estimates.

First, subtracting equation (7) for the elastic response from equation (17c), we restate it in the equivalent form

(18) $$\int_{\Omega_{c}}\left(\sigma^{\left(m\right)}-\sigma^{E}\right):\varepsilon \left(\bar{u}\right)\;\mathrm{dx}=0$$

for all $\bar{u}\in H^{1}\left(\Omega_{c};{\mathbb{R}}^{d}\right)$, such that $\bar{u}=0$ at $\Gamma_{D}$. After differentiation of the Dirichlet condition (equation (17b)) with respect to time, we have $u^{\left(m\right)}+\alpha {\dot{u}}^{\left(m\right)}=0$ at $\Gamma_{D}$ and insert it in equation (18) as the test function $\bar{u}$, resulting in the identity

(19) $$\int_{\Omega_{c}}\left(\sigma^{\left(m\right)}-\sigma^{E}\right):\left(\varepsilon \left(u^{\left(m\right)}\right)+\alpha \varepsilon \left({\dot{u}}^{\left(m\right)}\right)\right)\;\mathrm{dx}=0.$$

Substituting the test function $\bar{\sigma }=\sigma^{\left(m\right)}-\sigma^{E}$ into equation (17d), such that

$$\int_{\Omega_{c}}\left(\varepsilon \left(u^{\left(m\right)}\right)+\alpha \varepsilon \left({\dot{u}}^{\left(m\right)}\right)\right):\left(\sigma^{\left(m\right)}-\sigma^{E}\right)\;\mathrm{dx}=\int_{\Omega_{c}}\left(F\left(\sigma^{\left(m-1\right)}\right)+\delta \sigma^{\left(m\right)}\right):\left(\sigma^{\left(m\right)}-\sigma^{E}\right)\;\mathrm{dx},$$

the left-hand side of this equality is zero, owing to equation (19); hence, we rearrange it as follows:

(20) $$\int_{\Omega_{c}}\delta ∥\sigma^{\left(m\right)}{∥}^{2}\;\mathrm{dx}=\int_{\Omega_{c}}\left(\delta \sigma^{\left(m\right)}:\sigma^{E}-F\left(\sigma^{\left(m-1\right)}\right):\left(\sigma^{\left(m\right)}-\sigma^{E}\right)\right)\;\mathrm{dx}.$$

Applying the Young and Cauchy–Schwartz inequalities to the right-hand side of equation (20) and using the upper bound of F from equation (5a) provides us with the inequality

(21a) $$\frac{\delta }{2}\int_{\Omega_{c}}∥\sigma^{\left(m\right)}{∥}^{2}\;\mathrm{dx}\leq \int_{\Omega_{c}}\left(\frac{M_{1}^{2}}{\delta }+\delta ∥\sigma^{E}{∥}^{2}+M_{1}∥\sigma^{E}∥\right)\;\mathrm{dx}=:K_{4}.$$

Next, inserting $\bar{\sigma }=\varepsilon \left(u^{\left(m\right)}\right)$ as a test function in equation (17d) such that

$$\int_{\Omega_{c}}\left(∥\varepsilon \left(u^{\left(m\right)}\right){∥}^{2}+\frac{\alpha }{2}\frac{d}{\mathrm{dt}}∥\varepsilon \left(u^{\left(m\right)}\right){∥}^{2}\right)\;\mathrm{dx}=\int_{\Omega_{c}}\left(F\left(\sigma^{\left(m-1\right)}\right)+\delta \sigma^{\left(m\right)}\right):\varepsilon \left(u^{\left(m\right)}\right)\;\mathrm{dx},$$

integrating the result over time t∈(0,τ) with the help of the initial condition (equation (17a)), and applying the Young inequality to its right-hand side, it follows that

$$\begin{array}{c} \frac{1}{2}\int_{Q_{c}^{\tau }}∥\varepsilon \left(u^{\left(m\right)}\right){∥}^{2}\;\mathrm{dx}\mathrm{dt}+\frac{\alpha }{2}\int_{\Omega_{c}}∥\varepsilon \left(u^{\left(m\right)}\right)\left(\tau \right){∥}^{2}\;\mathrm{dx}\\ \leq \frac{\alpha }{2}\int_{\Omega_{c}}∥\varepsilon \left(u^{0}\right){∥}^{2}\;\mathrm{dx}+\int_{Q_{c}^{\tau }}\left(∥F\left(\sigma^{\left(m-1\right)}\right){∥}^{2}+\delta^{2}∥\sigma^{\left(m\right)}{∥}^{2}\right)\;\mathrm{dx}\mathrm{dt}. \end{array}$$

Taking the maximum over τ∈[0,T] here, using the upper bounds of F from equation (5a), and $\sigma^{\left(m\right)}$ from equation (21a), we derive the second estimate

(21b) $$\begin{array}{c} \frac{1}{2}\int_{Q_{c}^{T}}∥\varepsilon \left(u^{\left(m\right)}\right){∥}^{2}\;\mathrm{dx}\mathrm{dt}+\frac{\alpha }{2}\underset{t\in \left[0,T\right]}{\max}\int_{\Omega_{c}}∥\varepsilon \left(u^{\left(m\right)}\right){∥}^{2}\;\mathrm{dx}\\ \leq \frac{\alpha }{2}\int_{\Omega_{c}}∥\varepsilon \left(u^{0}\right){∥}^{2}\;\mathrm{dx}+M_{1}^{2}|Q_{c}^{T}|+2\mathrm{\delta T}K_{4}=:K_{5}. \end{array}$$

Finally, inserting $\bar{\sigma }=\alpha \varepsilon \left({\dot{u}}^{\left(m\right)}\right)$ as a test function in equation (17d), it follows that

$$\alpha^{2}\int_{\Omega_{c}}∥\varepsilon \left({\dot{u}}^{\left(m\right)}\right){∥}^{2}\;\mathrm{dx}=\int_{\Omega_{c}}\left(F\left(\sigma^{\left(m-1\right)}\right)+\delta \sigma^{\left(m\right)}-\varepsilon \left(u^{\left(m\right)}\right)\right):\alpha \varepsilon \left({\dot{u}}^{\left(m\right)}\right)\;\mathrm{dx}.$$

Applying the Young and Cauchy–Schwartz inequalities to the right-hand side here, with the help of the upper bounds of F in equation (5a), $\sigma^{\left(m\right)}$ in equation (21a), and $\varepsilon \left(u^{\left(m\right)}\right)$ in equation (21b), we get

(21c) $$\frac{\alpha^{2}}{4}\int_{\Omega_{c}}∥\varepsilon \left({\dot{u}}^{\left(m\right)}\right){∥}^{2}\;\mathrm{dx}\leq M_{1}^{2}|\Omega_{c}|+2\delta K_{4}+\frac{2K_{5}}{\alpha }=:K_{6}.$$

From the uniform estimates of equation (21), we conclude that the following mapping is well defined by equation (17) (see Dautray and Lions [26], Remark 1, p.555):

(22) $$m:C(\left[0,T\right];L^{2}\left(\Omega_{c};\mathrm{Sym}\left({\mathbb{R}}^{d\times d}\right)\right)\mapsto C(\left[0,T\right];L^{2}(\Omega_{c};\mathrm{Sym}\left({\mathbb{R}}^{d\times d},)\right),\;\sigma^{\left(m-1\right)}\mapsto \sigma^{\left(m\right)},$$

and by equation (21a) it maps the sphere

$$\left\{\sigma :\int_{\Omega_{c}}∥\sigma {∥}^{2}\;\mathrm{dx}\mathrm{dt}\leq \frac{2K_{4}}{\delta }\right\}$$

into itself.

The mapping m is continuous in the weak topology, that is provided by the weak-to-weak continuity of the nonlinear term F in equation (17d). Indeed, if

(23a) $$\sigma^{\left(m\right)}⇀\sigma^{\delta }\;\mathrm{weakly in}\;C(\left[0,T\right];L^{2}\left(\Omega_{c};\mathrm{Sym}\left({\mathbb{R}}^{d\times d}\right)\right),$$

then $u^{\left(m\right)}$, $\varepsilon \left(u^{\left(m\right)}\right)$, and $\varepsilon \left({\dot{u}}^{\left(m\right)}\right)$ also converge weakly to some $u^{\delta }$, $\varepsilon \left(u^{\delta }\right)$, and $\varepsilon \left({\dot{u}}^{\delta }\right)$, respectively, as a result of the uniform estimates of equations (21b) and (21c). Since equation (5a) implies the boundedness of $F\left(\sigma^{\left(m\right)}\right)$ in $C(\left[0,T\right];L^{\infty }\left(\Omega_{c};\mathrm{Sym}\left({\mathbb{R}}^{d\times d}\right)\right)$, there exists a convergent subsequence, still denoted by *m*, such that

(23b) $$F\left(\sigma^{\left(m\right)}\right)\to F^{\delta }\;\mathrm{strongly in}\;C(\left[0,T\right];L^{2}\left(\Omega_{c};\mathrm{Sym}\left({\mathbb{R}}^{d\times d}\right)\right),$$

and $F^{\delta }=F\left(\sigma^{\delta }\right)$ by Minty’s argument, as follows. Using the monotone property of equation (5b) and the convergences expressed in equation (23), we have for arbitrary $\bar{\sigma }\in L^{2}\left(\Omega_{c};\mathrm{Sym}\left({\mathbb{R}}^{d\times d}\right)\right)$ that

$$0\leq \underset{m\to \infty }{\lim}\int_{\Omega_{c}}\left(F\left(\sigma^{\left(m\right)}\right)-F\left(\bar{\sigma }\right)\right):\left(\sigma^{\left(m\right)}-\bar{\sigma }\right)\;\mathrm{dx}=\int_{\Omega_{c}}\left(F^{\delta }-F\left(\bar{\sigma }\right)\right):\left(\sigma^{\delta }-\bar{\sigma }\right)\;\mathrm{dx}.$$

Inserting here the test function $\bar{\sigma }=\sigma^{\delta }\pm s\tilde{\sigma }$ and dividing the result by *s* provides the inequality

$$\mp \int_{\Omega_{c}}\left(F^{\delta }-F\left(\sigma^{\delta }\pm s\tilde{\sigma }\right)\right):\tilde{\sigma }\;\mathrm{dx}\geq 0$$

that holds for arbitrary $\tilde{\sigma }\in L^{2}\left(\Omega_{c};\mathrm{Sym}\left({\mathbb{R}}^{d\times d}\right)\right)$. Passing to the limit as s→0 based on the hemi-continuity property (equation (5c)) leads to the equality $F^{\delta }=F\left(\sigma^{\delta }\right)$ in equation (23b) and hence proves the continuity of the mapping m.

The Schauder–Tychonoff theorem, applied to the mapping m in equation (22), guarantees the existence of a fixed point. After passage of the linearized problem of equation (17) to the limit as m→∞, the fixed point $\left(u^{\delta },\varepsilon \left(u^{\delta }\right),\sigma^{\delta }\right)$ implies a solution of the regularized problem of equation (15), depending on δ as a parameter.

It remains to prove three a-priori estimates claimed in equation (16).

With the help of equation (7), the equilibrium equation (15c) is restated in a form similar to equation (18):

(24) $$\int_{\Omega_{c}}\left(\sigma^{\delta }-\sigma^{E}\right):\varepsilon \left(\bar{u}\right)\;\mathrm{dx}=0.$$

The differentiation of the Dirichlet condition (equation (15b)) with respect to time allows us to insert the test function $\bar{u}=u^{\delta }+\alpha {\dot{u}}^{\delta }$ in equation (24), resulting in the identity

(25) $$\int_{\Omega_{c}}\left(\sigma^{\delta }-\sigma^{E}\right):\left(\varepsilon \left(u^{\delta }\right)+\alpha \varepsilon \left({\dot{u}}^{\delta }\right)\right)\;\mathrm{dx}=0.$$

Substituting this into equation (15d) written for the test function $\bar{\sigma }=\sigma^{\delta }-\sigma^{E}$, similarly to equation (20), implies that

(26) $$\int_{\Omega_{c}}\left(\delta ∥\sigma^{\delta }{∥}^{2}+F\left(\sigma^{\delta }\right):\sigma^{\delta }\right)\;\mathrm{dx}=\int_{\Omega_{c}}\left(\delta \sigma^{\delta }+F\left(\sigma^{\delta }\right)\right):\sigma^{E}\;\mathrm{dx}.$$

Applying the Young and Cauchy–Schwartz inequalities to the right-hand side of equation (26), with the help of both the upper (equation (5a)) and lower (equation (5d)) bounds of F, results in the estimate of equation (16a). We note that equation (16a) is advantageous compared with equation (21a), since its left-hand side contains the *L*^1^ -norm of $\sigma^{\delta }$, independent of the regularization parameter δ. The proof of the estimates of equations (16b) and (16c) follows word-for-word the derivation of equations (21b) and (21c). Theorem 1 is proved. □

Based on the uniform estimates of equation (16), we pass the regularized problem of equation (15) to the limit as δ→0.

**Theorem 2. (i)** *Let the assumptions of Theorem 1 hold. There exists an accumulation point* (u,ε(u),σ) *of the solutions* $\left(u^{\delta },\varepsilon \left(u^{\delta }\right),\sigma^{\delta }\right)$ *of the regularized problem of equation (15) as* δ→0 . *It satisfies the initial (equation (9c)) and Dirichlet (equation (9d)) conditions, the variational equation (14a), and inequality* (14b).

**(ii)** *If the stress component of the solution is regular, such that* $\sigma \in C(\left[0,T\right];L^{2}\left(\Omega_{c};\mathrm{Sym}\left({\mathbb{R}}^{d\times d}\right)\right)$, *the triple* (u,ε(u),σ) *satisfies the weak formulation of equation (10). In this case, the following a-priori estimates hold*:

(27a) $$\begin{array}{cc} ∥\varepsilon \left(u\right){∥}^{2} & \leq \frac{1}{\alpha }M_{1}^{2}T+∥\varepsilon \left(u^{0}\right){∥}^{2}=:K_{7}, \end{array}$$

(27b) $$\begin{array}{cc} \alpha ∥\varepsilon \left(\dot{u}\right)∥ & \leq \sqrt{K_{7}}+M_{1}, \end{array}$$

(27c) $$\begin{array}{cc} M_{3}\int_{\Omega_{c}}\overset{d}{\underset{i,j=1}{\sum }}|\sigma_{\mathrm{ij}}|\;\mathrm{dx} & \leq M_{2}|\Omega_{c}|+M_{1}\int_{\Omega_{c}}∥\sigma^{E}∥\;\mathrm{dx}, \end{array}$$

*where an elastic stress* $\sigma^{E}$ *from equation (7) depends on the data f and g, and* $\varepsilon \left(u^{0}\right)\in L^{\infty }\left(\Omega_{c};\mathrm{Sym}\left({\mathbb{R}}^{d\times d}\right)\right)$ *is assumed. Moreover, if the monotone property (equation (5b)) of* F *is strict, the stress* σ *is unique.*

**Proof.** Since $L^{1}\left(\Omega_{c}\right)$ is not a reflexive space, we utilize its embedding in the space of bounded measures $M^{1}\left(\Omega_{c}\right)$ with the corresponding dual space $C_{c}\left(\Omega_{c}\right)$. From the uniform estimates of equation (16), we derive the existence of a subsequence, still denoted δ, such that the following convergences take place as δ→0, for every t∈[0,T] :

(28a) $$u^{\delta }⇀u\;\mathrm{weakly in}\;H^{1}\left(\Omega_{c};{\mathbb{R}}^{d},)\right),$$

(28b) $$\varepsilon \left(u^{\delta }\right)⇀\varepsilon \left(u\right),\;\varepsilon \left({\dot{u}}^{\delta }\right)⇀\varepsilon \left(\dot{u}\right)\;\mathrm{weakly in}\;L^{2}\left(\Omega_{c};\mathrm{Sym}\left({\mathbb{R}}^{d\times d}\right)\right),$$

(28c) $$\delta \sigma^{\delta }⇀0\;\mathrm{weakly in}\;L^{2}\left(\Omega_{c};\mathrm{Sym}\left({\mathbb{R}}^{d\times d}\right)\right),$$

(28d) $$\sigma^{\delta }⇀\sigma \;⋆-\mathrm{weakly in}\;M^{1}\left(\Omega_{c};\mathrm{Sym}\left({\mathbb{R}}^{d\times d}\right)\right).$$

By virtue of equation (28a), from equations (15a) and (15b), the initial (equation (9c)) and Dirichlet (equation (9d)) conditions follow. For a test function $\bar{u}\in H^{1}\left(\Omega_{c};{\mathbb{R}}^{d}\right)$, such that $\bar{u}=0$ at $\Gamma_{D}$ and $\varepsilon \left(\bar{u}\right)\in C_{c}\left(\Omega_{c};\mathrm{Sym}\left({\mathbb{R}}^{d\times d}\right)\right)$, passing equation (15c) to the limit as a consequence of equation (28d) leads to the variational equation (14a). For arbitrary $\tilde{\sigma }\in C_{c}\left(\Omega_{c};\mathrm{Sym}\left({\mathbb{R}}^{d\times d}\right)\right)$, substituting $\bar{\sigma }=\tilde{\sigma }-\sigma^{\delta }$ as a test function in equation (15d), we rearrange the result as follows:

$$\begin{array}{c} \int_{\Omega_{c}}\{\left(\varepsilon \left(u^{\delta }\right)+\alpha \varepsilon \left({\dot{u}}^{\delta }\right)\right):\tilde{\sigma }+\delta ∥\sigma^{\delta }{∥}^{2}-\delta \sigma^{\delta }:\tilde{\sigma }\}\;\mathrm{dx}\\ =\int_{\Omega_{c}}\left\{F\left(\sigma^{\delta }\right):\left(\tilde{\sigma }-\sigma^{\delta }\right)+\left(\varepsilon \left(u^{\delta }\right)+\alpha \varepsilon \left({\dot{u}}^{\delta }\right)\right):\sigma^{\delta }\right\}\;\mathrm{dx}. \end{array}$$

Using the non-negativeness of the term $\delta ∥\sigma^{\delta }{∥}^{2}$, the monotone property (equation (5b)) of F, and the variational equation (15c) with the test function $\bar{u}=u^{\delta }+\alpha {\dot{u}}^{\delta }$, we continue with the following inequality:

(29) $$\begin{array}{c} \int_{\Omega_{c}}\left\{\left(\varepsilon \left(u^{\delta }\right)+\alpha \varepsilon \left({\dot{u}}^{\delta }\right)\right):\tilde{\sigma }-\delta \sigma^{\delta }:\tilde{\sigma }\right\}\;\mathrm{dx}\\ \leq \int_{\Omega_{c}}F\left(\tilde{\sigma }\right):\left(\tilde{\sigma }-\sigma^{\delta }\right)\;\mathrm{dx}+\int_{\Omega_{c}}f\cdot \left(u^{\delta }+\alpha {\dot{u}}^{\delta }\right)\;\mathrm{dx}+\int_{\Gamma_{N}}g\cdot \left(u^{\delta }+\alpha {\dot{u}}^{\delta }\right)\;{\mathrm{dS}}_{x}. \end{array}$$

Passing equation (29) to the limit in virtue of the convergences established in equation (28), we arrive at the variational inequality (2).

Now we prove assertion (ii). Let $\sigma \in C(\left[0,T\right];L^{2}\left(\Omega_{c};\mathrm{Sym}\left({\mathbb{R}}^{d\times d}\right)\right)$. Since the space $C_{c}\left(\Omega_{c}\right)$ is dense in $L^{2}\left(\Omega_{c}\right)$, the variational equation (14a) turns into equation (10a), and inserting it in equation (2) leads to the inequality

(30) $$\int_{\Omega_{c}}\left(\varepsilon \left(u\right)+\alpha \varepsilon \left(\dot{u}\right)-F\left(\bar{\sigma }\right)\right):\left(\bar{\sigma }-\sigma \right)\;\mathrm{dx}\leq 0$$

which holds for all $\bar{\sigma }\in L^{2}\left(\Omega_{c};\mathrm{Sym}\left({\mathbb{R}}^{d\times d}\right)\right)$. Repeating Minty’s argument, for arbitrary $\tilde{\sigma }\in L^{2}\left(\Omega_{c};\mathrm{Sym}\left({\mathbb{R}}^{d\times d}\right)\right)$ and s>0, we can insert $\bar{\sigma }=\sigma \pm s\tilde{\sigma }$ as a test function in equation (30) and divide the result by *s*, such that

$$\pm \int_{\Omega_{c}}\left(\varepsilon \left(u\right)+\alpha \varepsilon \left(\dot{u}\right)-F\left(\sigma \pm s\tilde{\sigma }\right)\right):\tilde{\sigma }\;\mathrm{dx}\leq 0.$$

Taking the limit as s→0 with the help of the hemi-continuity property (equation (5c)) follows the variational equation (10b).

By the fundamental lemma of calculus of variation, the variational equation (10b) implies that the constitutive relation (9b) holds almost everywhere in $\Omega_{c}$, that is

(31) $$\varepsilon \left(u\right)+\alpha \varepsilon \left(\dot{u}\right)=F\left(\sigma \right),\;t\in \left(0,T\right).$$

Squaring both sides of equation (31), such that

$$∥\varepsilon \left(u\right){∥}^{2}+\alpha \frac{d}{\mathrm{dt}}\left(∥\varepsilon \left(u\right){∥}^{2}\right)+\alpha^{2}∥\varepsilon \left(\dot{u}\right){∥}^{2}=∥F\left(\sigma \right){∥}^{2},$$

integrating the result over t∈(0,τ) with the help of the initial condition (9c), and taking the maximum over τ∈[0,T], owing to the upper bound (equation (5a)) of F, we get the uniform estimate of ε(u) as follows:

$$\begin{array}{cc} \alpha ∥\varepsilon \left(u\right){∥}^{2}\leq \alpha \underset{t\in \left[0,T\right]}{\max}∥\varepsilon \left(u\right){∥}^{2}+\int_{0}^{T}\left(∥\varepsilon \left(u\right){∥}^{2}+\alpha^{2}∥\varepsilon \left(\dot{u}\right){∥}^{2}\right)\mathrm{dt} & =\int_{0}^{T}∥F\left(\sigma \right){∥}^{2}\;\mathrm{dt}+\alpha ∥\varepsilon \left(u^{0}\right){∥}^{2}\\ \leq M_{1}^{2}T+\alpha ∥\varepsilon \left(u^{0}\right){∥}^{2}, \end{array}$$

which implies equation (27a). The uniform estimate (equation (27b)) of $\varepsilon \left(\dot{u}\right)$ is the direct consequence of equations (5a), (27a), and (31), thus providing the inclusion $\varepsilon \left(u\right)\in C^{1}(\left[0,T\right];L^{\infty }\left(\Omega_{c};\mathrm{Sym}\left({\mathbb{R}}^{d\times d}\right)\right)$.

To prove the a-priori estimate (27c) for σ, we consider the auxiliary stress $\sigma^{E}$ corresponding to an elastic solution of equation (7), and insert $\bar{\sigma }=\sigma -\sigma^{E}$ as the test function in equation (10b), that is

$$\int_{\Omega_{c}}\left(\varepsilon \left(u\right)+\alpha \varepsilon \left(\dot{u}\right)\right):\left(\sigma -\sigma^{E}\right)\;\mathrm{dx}=\int_{\Omega_{c}}F\left(\sigma \right):\left(\sigma -\sigma^{E}\right)\;\mathrm{dx},$$

then substitute $\bar{u}=u+\alpha \dot{u}$ in equations (7) and (10a), leading to the identity

$$\int_{\Omega_{c}}\left(\sigma -\sigma^{E}\right):\left(\varepsilon \left(u\right)+\alpha \varepsilon \left(\dot{u}\right)\right)\;\mathrm{dx}=0,$$

which together imply that

(32) $$\int_{\Omega_{c}}F\left(\sigma \right):\sigma \;\mathrm{dx}=\int_{\Omega_{c}}F\left(\sigma \right):\sigma^{E}\;\mathrm{dx}.$$

Applying the lower bound (equation (5d)) to the left-hand side and the Cauchy–Schwartz inequality and the upper bound (equation (5a)) to the right-hand side of equation (32), the a-priori estimate (27c) follows.

Uniqueness of the stress component can be argued by appealing to the strict monotone property of F. Indeed, assuming two different variational solutions $\left(u^{1},\varepsilon \left(u^{1}\right),\sigma^{1}\right)$ and $\left(u^{2},\varepsilon \left(u^{2}\right),\sigma^{2}\right)$ of equation (10) leads to

$$\begin{array}{cc} \int_{\Omega_{c}}\left(\sigma^{1}-\sigma^{2}\right):\varepsilon \left(\bar{u}\right)\;\mathrm{dx} & =0, \end{array}$$

$$\begin{array}{cc} \int_{\Omega_{c}}\left(\varepsilon \left(u^{1}-u^{2}\right)+\alpha \varepsilon \left({\dot{u}}^{1}-{\dot{u}}^{2}\right)-F\left(\sigma^{1}\right)+F\left(\sigma^{2}\right)\right):\bar{\sigma }\;\mathrm{dx} & =0. \end{array}$$

Substituting $\bar{u}=u^{1}-u^{2}+\alpha \left({\dot{u}}^{1}-{\dot{u}}^{2}\right)$ and $\bar{\sigma }=\sigma^{1}-\sigma^{2}$ here, we get

$$\int_{\Omega_{c}}\left(F\left(\sigma^{1}\right)-F\left(\sigma^{2}\right)\right):\left(\sigma^{1}-\sigma^{2}\right)\;\mathrm{dx}=0.$$

If the inequality (5b) is strict for $\sigma =\sigma^{1}\neq \sigma^{2}=\bar{\sigma }$, this leads to a contradiction. Therefore, $\sigma^{1}=\sigma^{2}$ is unique. The proof is complete. □

## 4. Discussion

We note that we can set α=0 in equation (9), which results in the nonlinear elasticity problem with limiting small strain and the stress-free crack. In this particular case, the a-priori estimate (equation (16b)) can be improved within the proof of Theorem 1 as follows:

$$\frac{1}{2}\int_{\Omega_{c}}∥\varepsilon \left(u^{\delta }\right){∥}^{2}\;\mathrm{dx}\leq M_{1}^{2}|\Omega_{c}|+2\delta K_{1},$$

thus establishing the existence Theorem 2. The variational solution obeys the following evident property of boundedness of the strain tensor:

$$∥\varepsilon \left(u\right)∥=∥F\left(\sigma \right)∥\leq M_{1}$$

instead of equations (27a) and (27b). Moreover, the nonlinear crack problem subject to contacting crack faces was established in the work of Itou et al. [24].

To incorporate the contact conditions, the Kelvin–Voigt viscoelastic model could be relaxed within the creep context (following Khludnev and Kovtunenko [8], Section 3.1).
